# Food Insecurity and Insulin Use in Hyperglycemic Patients Presenting to the Emergency Department

**DOI:** 10.5811/westjem.2020.4.45918

**Published:** 2020-07-03

**Authors:** Heng Ky Nhoung, Munish Goyal, Maria Cacciapuoti, Hannah Day, Taymour Hashemzadeh, Michelle Magee, Yumi S. Jarris

**Affiliations:** * Georgetown University School of Medicine, Washington, District of Columbia; † MedStar Washington Hospital Center, Department of Emergency Medicine, Washington, District of Columbia; ‡ MedStar Health, MedStar Diabetes Institute, Columbia, Maryland; § MedStar Georgetown University Hospital, Georgetown University School of Medicine, Department of Family Medicine, Washington, District of Columbia

## Abstract

**Introduction:**

The prevalence of food insecurity (FI) and insulin rationing among patients with diabetes who present to the emergency department (ED) is unclear. We examined the prevalence of food insecurity and subtherapeutic insulin use among patients who presented to the ED with a blood glucose level of greater than 250 milligrams per deciliter.

**Methods:**

This was a single-center, cross-sectional survey of clinically stable, hyperglycemic adults in the ED for food insecurity using the Hunger Vital Sign screening tool. Patients who were insulin dependent were asked about insulin usage and rationing.

**Results:**

Of the 85 eligible patients, 76 (89.4%) were enrolled; 35 (46%) screened positive for food insecurity. Food insecure patients were 1.9 times more likely to be hospitalized than non-food insecure patients (relative risk = 1.90 [1.21–2.99], p<.01). Food insecure patients were younger than non-food insecure patients (50.4 vs 57.5 p<.02), and had significantly higher hemoglobin A1c (HgbA1c) levels (11.2% vs 9.9% p = 0.04). Of the 49 patients prescribed insulin, 17 (34.6%) stated they had used less insulin during the prior week than had been prescribed, and 21 (42.9%) stated they had used less insulin during the prior year than had been prescribed. Food insecure patients were more likely to have used less insulin than prescribed in the prior year (odds ratio = 3.60 [1.09–11.9], p = 0.04).

**Conclusion:**

Our exploratory findings suggest almost half of clinically stable adults presenting to our inner-city ED with hyperglycemia experience food insecurity. More than one-third of those prescribed insulin used less than their prescribed amount in the prior year.

## INTRODUCTION

Food insecurity (FI) is defined as “limited or uncertain availability of nutritionally adequate and safe foods or limited or uncertain ability to acquire acceptable foods in socially acceptable ways.”^1^ Previous research has suggested that food-insecure patients are more likely to have diabetes than those who are not food insecure, adjusting for other socioeconomic factors and physical activity.^2^ Those living with diabetes and food insecurity are at high risk of poor outcomes due to the struggle of deciding whether to spend limited financial resources on food or medication.^3^,^4^

Insulin prices have increased 300% in the past decade, leading to reports of patients rationing insulin.^5^ The prevalence of insulin rationing to save money has not been well described among the general diabetic population. Herkert et al reported their experience at the Yale Diabetes Center and found that 25.5% reported cost-related insulin underuse. Among these patients, more than one-third did not inform their physician.^6^

Existing data on the prevalence of FI are variable and are likely dependent on a number of factors including the clinical environment in which patients are surveyed. Data from DC Hunger Solutions demonstrated that 15% of the general population in Washington DC is food insecure.^7^ In a single-center study performed at a large, urban Minneapolis emergency department (ED), more than a fifth (22%) of patients were food insecure.^8^ Among diabetic patients in urban safety net clinics in San Francisco, 60% were food insecure.^9^ Currently there are no published data on the rate of FI among high-risk groups such as diabetic patients who present to the ED.

Efficiently screening for FI may provide an opportunity to mitigate this social determinant of health. In this study we aimed to quantify the prevalence of FI and subtherapeutic insulin usage among diabetic patients who present to the ED with hyperglycemia. We hypothesized that the prevalence of FI was higher among patients with hyperglycemia treated in the ED compared with the general population.

## METHODS

We conducted a single-center, cross-sectional exploratory study and report in accordance with the Strengthening the Reporting of Observational Studies in Epidemiology (STROBE) Guidelines.^10^ The study protocol was reviewed and approved by our institutional review board. Subjects were enrolled from June 11–July 26, 2019, between the hours of 8 am and 10 pm Monday through Friday at an urban, adult, tertiary care teaching hospital ED with ~ 90,000 annual visits. Trained and supervised research assistants were electronically notified when any patient in the ED had a blood glucose > 250 milligrams per deciliter (mg/dL) using a screening tool built into the electronic health record. After confirming medical stability with the clinical team, research assistants verbally consented subjects and provided an information sheet detailing involvement. Non-English speakers were excluded because we did not have the resources to reliably consent these patients.

**Figure 1:**
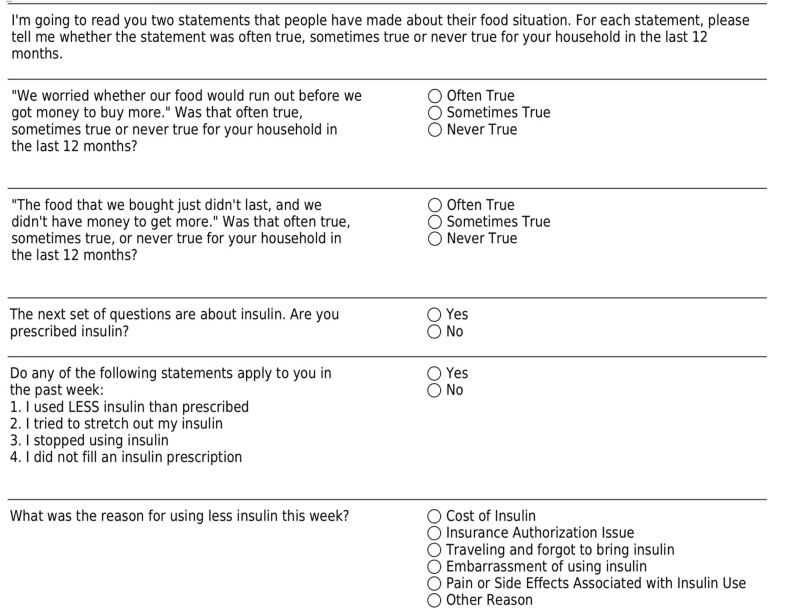
Questionnaire items including the Hunger Vital Sign and screen for insulin use.

Consenting subjects were verbally administered the previously validated Hunger Vital Sign screening tool for FI.^11^ A response from the patient with “Often True” or “Sometimes True” from either question was categorized as a positive screen. Insulin-dependent subjects were also asked questions regarding insulin rationing adapted from Herkert et al. (Figure 1).^6^ In addition, we recorded baseline characteristics (including age, gender, race/ethnicity, weight, height, pre-existing comorbid conditions); vital signs at presentation, pertinent laboratory variables (including basic metabolic panel, anion gap, beta-hydroxybutyrate, venous/arterial blood gas, urinalysis); years since diagnosed with diabetes; outpatient insulin regimen (insulin types, dose, timing); how many doses missed in the past week or month; and disposition from the ED. Data were collected via an encrypted, standardized, REDCap data collection tool. If patients screened positive for FI, they were given an information sheet of resources to contact for emergency food and other public services based on their ZIP Code of residence.

**Population Health Research Capsule**

What do we already know about this issue?

*Food insecurity (FI) is associated with poorer health outcomes. Screening for FI may provide an opportunity to mitigate this social determinant of health*.

What was the research question?

What is the prevalence of FI in hyperglycemic patients presenting to the emergency department (ED)?

What was the major finding of the study?

*Forty-six percent of patients presenting to our inner-city ED with hyperglycemia experienced FI*.

How does this improve population health?

*There are no known simple clinical markers for FI for screening in the ED. Hyperglycemia may be an objective marker for FI, but this data requires external validation*.

The primary outcome was percentage of patients who screened positive for FI in the past 12 months. Secondary outcomes included percentage of patients who reported using less insulin than prescribed in the past week and the percentage who reported using less insulin than prescribed in the past year. Patient characteristics were assessed with descriptive statistics and frequency distributions. We compared categorical variables using the chi-square test or Fisher’s exact test. Continuous variables were compared using the independent samples t-test or Wilcoxon’s rank-sum test.

## RESULTS

**Figure 2:**
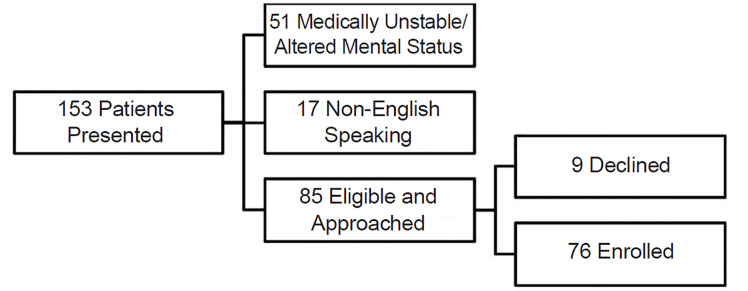
Patient enrollment in study of insulin use and food insecurity.

In total, 153 patients presented with a blood glucose greater than 250 mg/dL. Of those, 85 were eligible of whom nine declined, resulting in 76 subjects enrolled in our study (Figure 2). Mean age was 53.7 years, 58% were female, and 91.7% were Black. Of the 76 patients enrolled, two were homeless and two were housed in skilled nursing facilities. Refer to Table 1 for additional demographics. Of 76 subjects, 35 (46.1%) reported FI in the prior year.

Forty-nine of the 76 subjects reported they had been prescribed insulin. Of these, 34.7% (17 of 49) reported using less insulin than prescribed in the past week and 42.9% (21 of 49) in the past year. Three of the 17 subjects (17.6%) reporting using less insulin than prescribed in the past week due to cost. All of these subjects also reported FI (Table 2). Other reasons for not using insulin as prescribed included traveling and forgot insulin; undesirable side effects; and prescription filling/authorization issues. There was no majority in the reasons for patients reporting insulin underuse.

Hyperglycemic patients who reported FI were 1.9 times more likely to be admitted to the hospital than discharged than hyperglycemic patients who did not report being food insecure (Table 3).

## DISCUSSION

In this exploratory study, we sought to determine the prevalence of FI and insulin underuse in hyperglycemic patients in an urban ED. The prevalence of FI in our study is similar to that described in diabetic patients in safety net clinics in San Francisco,^9^ but more than double the rate described by Miner et al among all clinically stable patients presenting to their urban ED in Minneapolis.^8^ Furthermore, our study found that the rate of FI among those with a glucose > 250 mg/dL in our ED was triple the rate of the general population in Washington, DC, as reported by DC Hunger Solutions (15%) suggesting that those who use our ED and those who are hyperglycemic are more likely to be food insecure.^7^

Patients who screened positive for FI were significantly more likely to be admitted to the hospital and have significantly higher HgbA1c levels. These results suggest that those who are food insecure have worse control of their diabetes leading to complications and hospitalization. It is unclear how much FI contributes to the lack of control vs being associated with poor control. It is plausible that if one is unsure what and when their next meal may be, they may be less likely to use their prescribed dose of diabetic medication(s). It is also possible that those with FI may have fewer healthy food choices, exacerbating their glycemic control and contributing to the observation that food-insecure patients were almost twice as likely to be admitted than those with food security.

Many studies have looked at possible interventions to address FI, but little is known about the efficacy of these interventions. One study interviewed a group of food-insecure patients who received written or verbal information about local resources and interviewed another group of food-insecure patients who received active, clinic-guided enrollment with a food resource program.^9^ In this clinic-guided enrollment, clinic staff would complete the program application and connect the patient with the program. This clinic-guided method had a much higher success rate than sharing written or verbal information about local resources based on follow-up interviews (31% vs 0–4%).^9^ These data suggest that an intervention for food-insecure patients should include active support to enroll patients into food assistance programs.

FI is an important social determinant of health; it is insidious and has a profound impact on patient well-being. The ED may be the only point of care for many disadvantaged patients. There are no known simple clinical markers for FI that can be used for screening in the ED. The results of this study suggest that hyperglycemia may be an objective predictive clinical marker for FI. This hypothesis will need to be validated in a larger cohort of ED patients and among diverse ED settings. If our preliminary findings are validated, blood glucose levels, and specifically the presence of hyperglycemia, could be employed as a simple screening tool to identify FI, the next step in addressing this important social determinant of health.

## LIMITATIONS

There were several limitations to this observational study. Patients had to be clinically stable and awake to be screened, which could have biased the patient population to those who were healthier. Answers were patient reported; therefore they were subject to recall bias. Patients may have had reservations about answering that they indeed had FI, which may have led to under-reporting and a falsely low reported prevalence of FI. We used a prior study’s ED FI rate,^8^ and did not include non-hyperglycemic patients. It is possible the prevalence of FI is high in our ED population and may not be unique to hyperglycemic patients.

Results from this convenience sample may not be generalizable to populations that were excluded, including non-English speakers and those presenting overnight and on weekends who may have differed in their prevalence of FI. In addition, we did not record reason for presentation and, therefore, were unable to determine what portion of the study subjects presented with issues related to diabetes. Finally, our institution is a large, safety-net hospital as represented by more than half of responding participants noting an annual household income of ≤ $25,000. This may overestimate the prevalence of FI in locations with less poverty.

## CONCLUSION

Our data suggest almost half of clinically stable, hyperglycemic adults in our inner-city ED experience food insecurity. More than one-third of those prescribed insulin used less than prescribed for various reasons. The prevalence of food insecurity in an undifferentiated population and the use of hyperglycemia as a screening tool for food insecurity warrant further study.

## Figures and Tables

**Table 1:** Demographics.

	Food insecure	Non-food insecure	Total
Number of patients enrolled	35 (46.1%)	41	76
(Average Age ± SD) p=.05	(50.4 ± 12.6) n= 33	(56.5 ± 13.2) n=39	(53.7 ± 13.2) n=72
% Female	55%	60%	58%
Race (N=72)			
Black	29	37	66
Non-Hispanic White	1	1	2
Hispanic	1	1	2
Other	2	0	2
Annual income (N = 66)			
Less than $12,490	13	11	24
$12,490–$25,000	11	3	14
$25,000–$50,000	3	11	14
$50,000–$75,000	1	3	4
$75,000–$100,000	2	2	4
>$100,000	0	6	6
Education level (N = 73)			
High school/GED	20	28	48
Associates	5	2	7
Bachelors	3	5	8
Masters/Doctorate	2	3	5
Trade school	0	1	1
None of the above	3	1	4
Prescription Coverage Through Medicare, Medicaid or Private Health Insurance (N = 73)			
Coverage	32	36	68
No coverage	1	4	5
(Average HgbA1c ± SD) p = 0.04	(11.2 ± 1.9) n = 25	(9.9 ± 1.9) n = 15	(10.7 ± 2.0) n = 40

*SD*, standard deviation; *GED*, general education development.

**Table 2:** Insulin use in the past week and year.

	Used less insulin than prescribed in the past week		Used less insulin than prescribed in the past year	
	YES	NO	YES	NO
Food insecure	11	13	14	10
Non-food insecure	6	19	7	18
	OR =2.68 (0.79–9.07) p =0.11		OR = 3.60 (1.09–11.9) p = 0.04	

*OR*, odds ratio.

**Table 3:** Patient disposition: admitted to the hospital or discharged to home.

Disposition	Admitted to the hospital	Discharged to home
Food insecure (35)	25 (71.4%)	10 (28.6%)
Non-food insecure* (40)	15 (37.5%)	25 (62.5%)
RR = 1.90 (1.21–2.99), p<.01		

* One patient who was non-food insecure left against medical advice.

*RR*, relative risk.
